# Alteration in Expression of Primordial Germ Cell (PGC) Markers During Induction of Human Amniotic Mesenchymal Stem Cells (hAMSCs)

**Published:** 2020

**Authors:** Farzane Alifi, Hamid Reza Asgari

**Affiliations:** 1- Department of Anatomical Sciences, School of Medicine, Iran University of Medical Sciences, Tehran, Iran; 2- Cellular and Molecular Research Center, Iran University of Medical Sciences, Tehran, Iran

**Keywords:** Amnion, Cell differentiation, Germ cells, Mesenchymal stem cells, Retinoic acid

## Abstract

**Background:**

Currently, scientists are looking for a solution to the problem of the couples who have a lack of germ cells by through cell therapy. It is found that human amniotic membrane mesenchymal stem cells (hAMSCs) could be a good candidate for solving this problem. In the present study, an attempt was made to show that hUMSCs can express the PGC markers in the presence of retinoic acid (RA).

**Methods:**

Placenta was obtained from healthy mothers and amniotic stem cells were isolated by enzymatic method from amniotic membrane. The cells were treated by retinoic acid for 14 days. Mesenchymal properties of hAMSCs were assessed by flow-cytometry and expression of PGC markers was established by Q-PCR.

**Results:**

Mesenchymal stem cell properties were confirmed by antibodies against mesenchymal stem cell markers (CD73, CD90, and CD105). After that, the expression of the C-kit, Oct4, SSEA4, VASA genes were determined as primordial germ cell markers using quantitative PCR. It was found that the use of retinoic acid led to the highest expression of C-kit, SSEA4, VASA genes and lower expression of Oct4.

**Conclusion:**

Our study indicates that retinoic acid can be used as a suitable factor for induction of hAMSCs into primordial germ cells (PGCs) and hAMSCs have enough potential to do that.

## Introduction

Recently, many couples are suffering from infertility. The world health organization declares that infertility is the main problem for 12–15 percent of couples in the world. In addition, many people engaged with cancer and related treatments such as chemotherapy that lead to disorder in the generation of germ cells (1). Other people also suffer from disorders such as azoospermia. It is clear that lack of germ cells causes infertility in such people. Based on today’s knowledge, clinicians suggested different methods such as medical treatment, assisted reproductive technology (ART) with new techniques like *in vitro* fertilization (IVF) and intracytoplasmic sperm injection (ICSI) to get pregnant. However, these methods may be not effective for all couples because some of them suffer from lack of germ cells congenitally or due to secondary reason such as chemotherapy or radiotherapy (1).

Recently, cell therapy has been suggested for infertility treatment in these patients (2, 3). Scientists have worked to treat infertility through the induction of stem cells into the germ cells, male or female gametes in the laboratory (4, 5). These cells are used for *in vitro* fertilization and zygote generation for the patients (6, 7).

It is found that amniotic membrane has a lot of mesenchymal stem cells with high differentiation potential (8). Researchers identified that hAMSCs are high potential stem cells and can differentiate into tissues that represent all three embryonic layers and announced that these cells have a higher potential for growth and proliferation than adult mesenchymal stem cells (*e.g*. bone marrow stem cells) *in vitro* (9). Scientists isolated hAMSCs by enzymatic digestion method from amniotic membrane and showed that these cells are positive for mesenchymal markers, CD73, CD105 and negative for CD14 (Strongly positive in monocytes) and CD34 (Expressed on early hematopoietic stem cells) (10). The result of some studies shows that hAMSCs are pluripotent stem cells which can be differentiated into all embryonic cell types, *e.g*., ectodermal, endodermal and mesodermal cells (11).

Amniotic membrane (AM) is the inner layer of the placenta which contains a large number of mesenchymal stem cells, has self-renewal, high proliferation and differentiation capacity (12). Human amniotic mesenchymal stem cells (hAMSCs) have several benefits; they are conveniently available, low-priced, they do not target ethical issues and have very low expression of histocompatibility antigens (13). The hAMSCs are human originated and they have minimal heterogeneity and accordingly, their transplantation will have minimal risk for the recipients (14).

Retinoic acid is an oxidized form of vitamin A, that is naturally produced in gonadal ridge and plays a key role in regulating post-migratory germ cell decision-making in order to enter the meiosis or stop at the mitosis stage (15). Scientists worked on several types of stem cells from different sources containing various characteristics. Mesenchymal stem cells were isolated from bone marrow by Friendestein and his colleagues for the first time in 1976 (16). Another researcher used mouse embryonic stem cells and induced them into oocyte-like cells (17). Some scientists studied mouse bone marrow mesenchymal stem cells and tried to direct them into male germ cells, in the presence of higher doses of retinoic acid (18). Another study has indicated that human umbilical cord mesenchymal stem cells could differentiate into male germ-like cells using retinoic acid during 14 days (19).

The novelty of this study is the use of human stem cells from amniotic membrane associated with high differentiation potential and an attempt was made to indicate the changes in expression of PGC markers after induction of these cells (hAMSCs) by retinoic acid within 14 days.

## Methods

### Isolation and culture of hAMSCs:

Our experimental procedures for sampling human placenta were performed according to ethical approvals by Iran University of Medical Sciences. Regarding the above ethical issues, with the assistance of the gynecologist at the Imam Hossein hospital, special consent forms were signed by the five mothers and after the birth of a healthy newborn by cesarean section, the placenta was transferred to our laboratory in a sterile container containing HBSS (Hanks Balanced Salt Solution) on ice.

After washing the placenta with PBS, using a blade and sterile forceps, the amniotic membrane was separated from the chorionic plate and the amniotic membrane was cut into small pieces. For isolation of human amniotic mesenchymal stem cells (hAMSCs), amniotic membrane was incubated in 0.05% collagenase type 1 and 4 for 60 *min* and Dispase 2 (2.5 *mg/ml*, Invitrogen) for 7 *min* at 37*°C*. To remove tissue remnants, cell suspension was filtered through 100 *μM* cell strainer. Then, cells were cultured in DMEM-F12 supplemented with 10% FBS and 100 *units/ml* penicillin-streptomycin and maintained in 37*°C* humidified incubator with 5% CO_2_. The culture medium was changed every 2–3 days. Amniotic cells were passaged by 0.25% Trypsin-EDTA and morphologically evaluated with an inverted phase contrast microscope (Olympus CKX-41). Cells upon 80 to 90% confluence were passaged and the medium was refreshed every other day. Cells from passages 3 to 4 were used for differentiation studies. hAMSCs were cultured in 3 groups. For each group, three 25 *cm*^2^ culture flasks were prepared at a density of 50000 *cells/cm*^2^.

### Flow-cytometry analysis:

In order to illustrate the purity of hAMSCs, the cell surface markers were analyzed by flow-cytometry. hAMSCs at 3rd passage, after washing with PBS (Phosphate buffered saline) were passaged by 0.25% trypsin-EDTA solution and centrifuged at 1000 *rpm* for 5 *min*. After discharging the supernatant, the pellet was washed two times with PBS. Then 50 *μl* of cell suspension were transferred to 6 tubes for an isotype control and tests. After fixation with the 1% paraformaldehyde solution, these aliquots were incubated with monoclonal antibodies against CD34- PE, CD45-FITC, CD73-PE, CD90-FITC, CD105-F ITC, and mouse IgG-FITC/PE for 30 *min* at 4*°C* in the dark. For cell surface staining, isotype antibodies conjugated to FITC or PE were used. Finally, the cells were analyzed using a Partec PAS III flow-cytometry system with Flomax software.

### hAMSCs differentiation and experimental groups:

To induce differentiation into germ cells, hAMSCs at 3rd passage were treated with 1 and 2 *μM* all-trans retinoic acid (Sigma-Aldrich) for 14 days. The control group (Control) was received culture medium without retinoic acid. The culture was repeated three times for each group. Culture medium contained DMEM-F12 supplemented with 5% FBS (FBS–GIBCO). Half of this medium was refreshed every 2 to 3 days and the culture continued for 14 days.

### Real-time RT-PCR:

This process was performed based on the method used in our previous reports (20).

Real time RT-PCR was performed to distinguish changes in the expression level of germ cell specific markers; Oct4, VASA, SSEA4, and C-kit— in the treated hAMSCs. Briefly, RNA extraction was performed using TriPure reagent (Roche) according to the manufacturer’s instructions. RNase-free DNase I (Thermo Scientific, Waltham, MA) was used for the elimination of genomic DNA contamination for 30 *min* at 37*°C*. RNA concentration and sterility were assessed by spectrophotometric method (WPA spectrophotometer, Biochrom, UK). RNA was reverse transcribed into complementary DNA (cDNA) using a random hexamer and 1000 *ng* of DNA-free RNA using Transcriptor First Strand cDNA Synthesis Kit (Roche). TaqMan® Gene Expression Assays (Life Technologies) were conscripted to check the expression of SSEA-4, Oct4, Vasa, and C-kit, which were normalized in 18 *s* expression as a housekeeping gene (β-actin). The ingredients of PCR reaction for assessing gene expression included 10 *μl* TaqMan® Universal Master Mix, 1 *μl* TaqMan® Assay reagent, 0.5 *μl* (25 *ng*) cDNA and 8.5 *μl* distilled water. PCR cycling parameters were set for 10 *min* at 95*°C* (Polymerase activation) and 40 cycles of 95*°C* for 15 *s* and 60*°C* for 1 *min* using a Rotor-Gene Q instrument (Qiagen). Using the ΔΔCt method, the relative expression of targets was computed by normalizing CT values of targets in 18 *s*. Each reaction was identically replicated (Usually three times) for each sample.

## Results

### Cell morphology:

**Figure 1.**
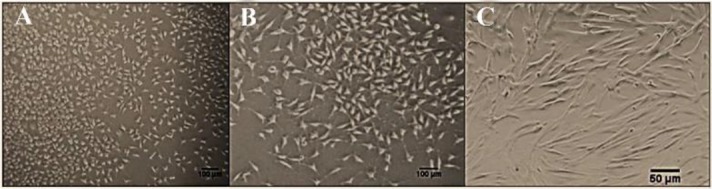
Human amniotic mesenchymal stem cells in the culture. The cells at the third passage, almost all of the cells were elongated and spindle-shaped. A) Magnification 100×. B) Magnification 200×. C) Magnification, 400×

After 5–7 days of culture, the hAMSCs were attached to the bottom of the flasks, the medium was changed every three days until cells covered the bottom of flask completely. After the first passage, most of the cells revealed spindle-shaped morphology, similar to fibroblasts and had processes in different sizes. Our cell culture was continued to the 3rd passage and a large number of hAMSCs could be observed which covered the bottom of the flasks (Figure 1A–C).

### Flow cytometry findings:

**Figure 2.**
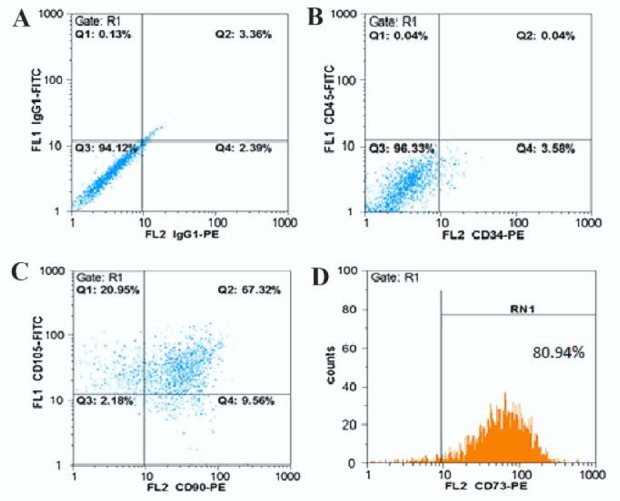
Characteristics of human amniotic mesenchymal stem cells (HAMSCs) by flow-cytometric analysis of surface-markers expression. The cells after passage 3 were labeled with PE or FITC-conjugated antibodies. A) The expression of IgG1-FITC as control marker, B) The negative expression of CD45 and CD34, and positive expression of C) CD105 and CD90, and D) CD73

hAMSCs at the third passage were evaluated by flow-cytometry to distinguish the mesenchymal stem cell characteristics. These cells were frequently positive against mesenchymal stem cell markers CD73, CD90, and CD105 and they minimally responded to CD34 (Endothelial marker) and CD45 (Hematopoietic markers). hAMSCs were 80.94%, 77.60% and 89.60% positive for CD73, CD90 and CD105, respectively. The cells also showed an insignificant presence for CD45 (0.04%) and CD34 (3.58%) (Figure 2A–D).

### Expression of primordial germ cell genes:

**Figure 3.**
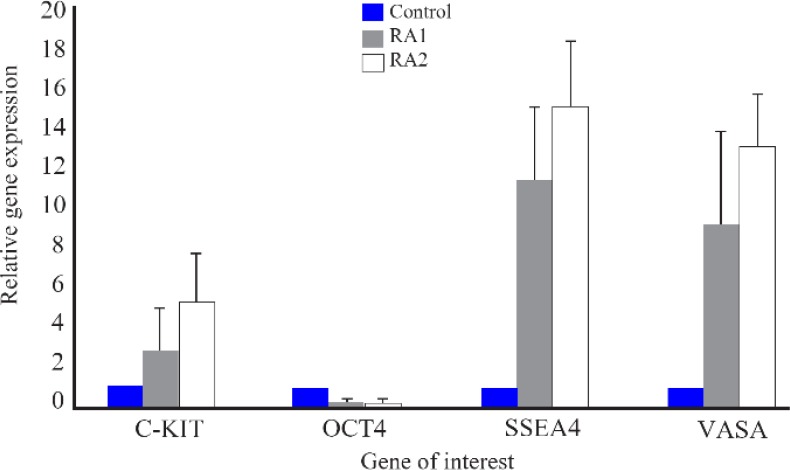
Changes in the expression of primordial germ cell (PGC) -specific markers in the human amniotic membrane mesenchymal stem cells (HAMSCs) after 14 days of treatment with 1 and 2 *μM* retinoic acid in comparison with control group. Values are shown as mean ±SD; n=3

The expression levels of PGC specific genes C-kit, Oct4, SSEA4, VASA with Q-PCR were evaluated in the control and treatment groups. Results showed that, hAMSCs after 14 days treatment with retinoic acid had significantly increased the expression of PGC specific genes. the treatment group with the 1 *μM* retinoic could up regulate 2.8, 0.233, 11.33, 9.21 times of C-kit, Oct4, SSEA4 and VASA genes up-regulation respectively, relative to the control group and treatment with 2 *μM* retinoic acid culminates in 5.3, 0.277, 14.92, 13.07 times of above-mentioned genes amplification respectively, relative to the control group (figure 3). As a result treatment with higher doses of retinoic acid was led to the highest expression of above genes except Oct4.

## Discussion

Our study showed that stem cells from the human amniotic membrane can express PGC markers by using retinoic acid within 14 days *in vitro*. Recent development in regenerative medicine and cell therapy has opened a new way in treatment of infertility by differentiation of stem cells in laboratories (20, 21). hAMSCs which originated from the extra-embryonic mesoderm belonging to fetal group of stem cells are proficient for this purpose because of their immunoprivileged status. Obviously, they express low levels of MHC-I and very low levels of MHC II which is the reason that hAMSCs could be suitable for human transplantation (22).

hAMSCs are fibroblast-like cells and capable for expression of mesenchymal stem cell markers such as CD73, CD90, and CD105 and lack of expression of CD34 and CD45 (Hematopoietic markers); these findings were previously reported by Kim (23). In previous studies, scientists benefited from 1 *μM* RA for inducing MSCs into PGCs and reported the expression of PGC markers within 15 days (24). Geijsen et al. (2004) indicated that RA has positive effects on the differentiation of early germ cells. They cultured embryonic stem cells for 7 days in the presence of RA and achieved PGCs and it means that RA has been effective for induction of embryonic stem cells in a short time. In our study, the positive effects of RA on induction of hAMSCs into PGCs were observed in a longer period of time, because the purpose was to assay late PGC markers (25). Nayernia et al. (2006) used bone marrow MSCs and treated them by 1 *μM* RA for 7, 14, 21 days, and succeeded to determine the specific PGC markers such as Vasa and Oct4, and reported that most of the colonies were created in 14 days (18). Similar to our experiment and in another study, efficiency of RA for inducting MSCs into germ cells during 21 days was established (26). As shown in the present study, 14 days usage of retinoic acid caused amplification of expression of germ cell genes significantly, while results of other study show that induction of Wharton’s jelly MSCs into germ cells needed 21 days, which is a long time for induction (27). Other researchers induced MSCs into male germ cells by using a mixture of RA and BMP4 and a co-culture system during 3 weeks. In presence of these two inducers and after 3 weeks, they achieved limited success; however, our results were obtained after 14 days (28).

It is important to know that higher concentrations of retinoic acid can endanger the cells in the culture system (29). Eguizabal et al. also used 2 *μM* retinoic acid for induction of embryonic stem cells into PGCs; in this study, also the same dose of RA was used (30). They could not achieve effective result by lower doses of RA. Since hAMSCs differentiation potential is higher than other adult MSCs, they need lower doses of RA for induction into germ-like cells (3, 31).

The expression of C-kit, Oct4, SSEA4, and VASA genes was assessed for confirmation of the differentiation of hAMSCs into PGCs, and it was found that RA can increase the expression of above genes, except Oct4, within 14 days. The above results are similar to the results of a group of scientists who found such outcomes on the mouse (21).

Oct4 is a pluripotency marker that in our study has been reduced. Its expression reduction indicates the beginning of differentiation of hAMSCs to germ cells and the decrease in the pluripotency properties (32). SSEA_4_ is a marker of pluripotency as well (33) and VASA gene, also called Ddx_4_, is a marker to identify germ cells and plays important role in the gametogenesis and germ cell development (34). C-kit gene has a key role in cell migration and survival of early germ cells (35). In this experimental study, retinoic acid was used in order to induce hAMSCs into PGCs within 14 days. It is found that retinoic acid promotes higher expression of VASA, C-kit, and SSEA4 genes and lower amounts of Oct4.

The distinct difference of this study can be seen in the usage of human stem cells and if required, these cells can easily be transplanted in the clinic without any immunological trick. Expression of the PGC markers from human amniotic stem cells is the first step in achieving the germ cells in the laboratory and could have a remarkable effect on infertility treatment. More research is needed to open up a new way to produce male and female germ cells to overcome infertility problems.

## Conclusion

Finding the appropriate cell for using in the human model and effective inducer for differentiating stem cells into germ cells is challenging. This study demonstrates that 2 *μM* as an optimal concentration of retinoic acid can be used for induction of hAMSCs into primordial germ cells (PGCs) *in vitro*.
